# Identification of cerebrospinal fluid biomarker candidates for anti-N-methyl-D-aspartate receptor encephalitis: High-throughput proteomic investigation

**DOI:** 10.3389/fimmu.2022.971659

**Published:** 2022-10-26

**Authors:** Yuchen Li, Keyu Yang, Fang Zhang, Jing Wang, Huijun Shen, Miaomiao Liu, Junhong Guo, Jie Wang

**Affiliations:** ^1^ Department of Neurology, First Hospital of Shanxi Medical University, Taiyuan, China; ^2^ Department of Critical Care Medicine, Aerospace Center Hospital, Beijing, China

**Keywords:** anti-N-methyl-D-aspartate receptor encephalitis, cytokines, biomarkers, cerebrospinal fluid, proteomics

## Abstract

**Background:**

Although the diagnosis is mainly dependent on the detection of anti-N-methyl-D-aspartate receptor (NMDAR) antibodies in cerebrospinal fluid (CSF) and/or serum, there was no direct correlations between anti-NMDAR antibody titers in CSF and disease severity and prognosis in anti-NMDAR encephalitis patients. Here, we aimed to extensively identify CSF biomarkers related to the occurrence, development, and prognosis of anti-NMDAR encephalitis using a high-throughput proteomic approach.

**Methods:**

A CSF cytokine antibody array containing 80 cytokines and inflammatory mediators related to immune and inflammatory responses was applied to identify biomarker candidates in individual CSF samples from a well-characterized cohort comprising patients with anti-NMDAR encephalitis (*n* = 6) and controls (*n* = 6). Validation and specific detection were performed in an extended cohort consisting of anti-NMDAR encephalitis patients (*n* = 13), controls (*n* = 13), and viral encephalitis (*n* = 13) by enzyme-linked immunosorbent assay (ELISA). Additionally, the levels of some inflammatory proteins in three groups in cohort 2 reported in previous literatures that may be involved in the development of anti-NMDAR encephalitis were also tested by ELISA. Correlations between candidate biomarkers and clinical characteristics of anti-NMDAR encephalitis patients were analyzed.

**Results:**

Three differentially expressed cytokines and inflammatory mediators were screened from the 80-cytokine array in cohort 1. Functional enrichment analysis results suggested that these differentially expressed proteins were related to autophagy, immune/inflammatory responses, cell death, and other processes. In cohort 2, the elevations of cellular inhibitor of apoptosis protein-1 (cIAP-1), macrophage colony-stimulating factor (MCSF), CXC chemokine ligand 13 (CXCL13), and nucleotide binding oligomerization domain-like receptor protein 3 (NLRP3) in anti-NMDAR encephalitis were validated by ELISA. Linear regression revealed that the levels of CSF CXCL13 and cIAP-1 were positively correlated with the highest modified Rankin scale (mRS) score in the acute phase (*p* < 0.05). The level of cIAP-1 was positively correlated with the anti-NMDAR Encephalitis One-Year Functional Status (NEOS) score (*p* < 0.05).

**Conclusion:**

These biomarkers show promising functions to evaluate severity or prognosis of anti-NMDAR encephalitis. The biological processes of immune/inflammatory responses, altered levels of autophagy, and the tumor necrosis factor (TNF) signal pathway may be involved in the pathophysiology of anti-NMDAR encephalitis to some extent.

## Introduction

Encephalitis with autoantibodies against the N-methyl-D-aspartate receptor (NMDAR) is the most common autoimmune encephalitis, which is characterized by a series of complex clinical neuropsychiatric symptoms (1). The diagnosis is mainly reliant on the positive result of antibodies in cerebrospinal fluid (CSF) or serum (2). The pathogenesis is not fully defined, and autoantibodies against NMDAR are currently considered critical factors. Dalmau et al. found that the effect of antibody on NMDARs density is titer-dependent; the higher the antibody titer, the greater the receptors’ density decreases, and the receptors’ density can gradually recover after antibody removal (2). Some clinical studies have suggested that the higher antibody titers have been associated with a more severe functional disability of anti-NMDAR encephalitis patients (3). These results suggested that antibody titers are correlated with the severity of NMDAR dysfunction and possibly with disease severity. However, recent studies have shown that there is no direct correlation between the anti-NMDAR antibody titer and the severity or prognosis of anti-NMDAR encephalitis patients, suggesting that other factors may play a role in the pathogenesis of anti-NMDAR encephalitis (3, 4). Therefore, further search for biomarkers related to disease is of important value for studying the pathogenesis of disease, evaluating the condition and judging the prognosis.

As important mediators that regulate the body’s immune responses, cytokines are involved in the occurrence of various immune diseases under certain conditions. More recently, studies have shown that some CSF cytokines participate in the pathogenesis and progression of anti-NMDAR encephalitis. Some studies have shown that inflammatory cytokines such as CXC chemokine ligand 13 (CXCL13) (5), nucleotide binding oligomerization domain-like receptor protein 3 (NLRP3) (6), interleukin 6 (IL-6), IL-17A, and IL-2 increased in CSF and/or serum of patients with anti-NMDAR encephalitis (7, 8), and are partly associated with poor prognosis, serious condition, the presence of infection aura, combined teratoma, and high recurrence rate. Some studies have pointed out that some inflammatory proteins that reflect CNS destruction also increased in CSF and/or serum of patients with anti-NMDAR encephalitis, such as neurofilament light chain (NFL) (9) and neuron-specific enolase (NSE) (10). These results have suggested that cytokines and some inflammatory mediators may play a role in the pathogenesis of anti-NMDAR encephalitis. However, the current analysis about cytokines and inflammatory mediator changes in anti-NMDAR encephalitis is limited. Most of the exploratory biomarker studies conducted in anti-NMDAR encephalitis follow a deductive reasoning to identify candidates based on existing knowledge of the pathophysiology of other autoimmune disorders. The functions of many inflammatory cytokines and inflammatory mediators in anti-NMDAR encephalitis remain unclear. Further extensive exploration and evaluation of the expressions of cytokines and inflammatory mediators in CSF of anti-NMDAR encephalitis patients and their possible roles can contribute to the screening of biomarkers as well as the exploration of pathological mechanisms.

This study aimed to screen out differentially expressed proteins between patients with anti-NMDAR encephalitis and patients without inflammatory diseases following an unbiased strategy employing proteomics. The differentially expressed proteins were verified by enzyme-linked immunosorbent assay (ELISA) in an expanded cohort. Considering that the clinical manifestations, EEG, and CSF cytology of anti-NMDAR encephalitis and viral encephalitis are similar, we added the viral encephalitis patients as control in validation cohort in order to find the cytokines or inflammatory mediators that may be used to distinguish the two diseases. The correlation analysis between differentially expressed proteins and clinical data of anti-NMDAR encephalitis patients was performed in order to identify candidate biomarkers for evaluation of the early diagnosis, prognosis, and therapeutic effect of anti-NMDAR encephalitis.

## Materials and methods

### Subject sample collection

Our study included anti-NMDAR encephalitis patients from the Department of Neurology, The First Hospital of Shanxi Medical University, Taiyuan, Shanxi, China, between January 2019 and December 2021. Inclusion criteria were as follows: (1) age ≥ 16 years; (2) cell-based analyses (CBAs) for CSF samples and serum samples before immunotherapy in the acute phase of symptoms onset; and (3) definite diagnosis of anti-NMDAR encephalitis using the criteria of Graus et al. (11). Exclusion criteria were as follows: (1) combination with other antibodies against neuronal and/or glial antigens; (2) complicated by central nervous system (CNS) infections and/or demyelinating diseases; and (3) suffering from other severe neurological or primary psychiatric complications (e.g., brain tumor, stroke, and myasthenia gravis).

The age- and sex-matched individuals with non-inflammatory diseases served as controls. The control group met the following criteria: (1) there were no obvious abnormalities in CSF cytology examination; (2) there were no definite or suspicious CNS infectious diseases; (3) there were no definite or suspected neuromyelitis optica spectrum disorder or multiple sclerosis; and (4) there were no definite or suspicious systemic autoimmune diseases. Patients with hydrocephalus, benign intracranial hypertension, hyperhomocysteinemia, and venous sinus thrombosis were mainly included.

All subjects underwent extensive clinical examinations including physical and neurological examinations, electroencephalogram (EEG), magnetic resonance imaging (MRI), and laboratory tests. The CSF samples were collected within 1 week after admission before immunotherapy. Anti-NMDAR antibody was detected in the Peking Union Medical College Hospital by a cell-based indirect immunofluorescence assay. We assessed their dominating clinical outcomes and prognosis on the ground of the modified Rankin scale (mRS) and anti-NMDAR Encephalitis One-Year Functional Status (NEOS) score (12). The study was performed according to the ethical principles of the Declaration of Helsinki and was approved by the local ethics committees. Written informed consent was obtained from all subjects.

### Phase 1: Screening differentially expressed cytokines and inflammatory mediators

#### Cohort 1

In this phase, six controls and six anti-NMDAR encephalitis patients were selected according to the criteria described above. The CSF of controls were numbered X10–X15, and the CSF of patients with anti-NMDAR encephalitis were numbered X30–X35. Differentially expressed cytokines and inflammatory mediators were screened from 80 cytokines and inflammatory mediators associated with immune and inflammatory responses by cytokine array analysis.

### Phase 2: Validation of candidate biomarkers

#### Cohort 2

The second part expanded the cohort size, including 13 patients with anti-NMDAR encephalitis and 13 controls. To obtain information on the specificity of the candidate biomarkers and to clarify the value of differentially expressed proteins in differentiating anti-NMDAR encephalitis from viral encephalitis, we analyzed 13 patients with viral encephalitis (VE) as another group of controls.

The viral encephalitis group met the following criteria: (1) the clinical manifestations were similar to those of anti-NMDAR encephalitis; (2) the virus infection in CSF was confirmed by mNGS, ELISA, or PCR; (3) there were no definite or suspected neuromyelitis optica spectrum disorder or multiple sclerosis; (4) negative autoimmune encephalitis-related antibody and good prognosis after antiviral treatment; and (5) there were no definite or suspicious systemic autoimmune diseases. The inclusion and exclusion criteria of the anti-NMDAR encephalitis group and control group were the same as before. The differentially expressed cytokines and inflammatory mediators screened in cohort 1 were validated. At the same time, the expression of some other candidate biomarkers [CXCL13 (5), NLRP3 (6), NFL (9), and NSE (10)] reported in previous literatures in CSF samples of three groups was detected and compared by ELISA.

### Cytokine array analysis

After completing the basic CSF analysis, such as cytological and biochemical examinations, the remaining CSF was centrifuged at 1,800 *g* at 4°C for 10 min, and the supernatant was aliquoted in 0.5-ml polypropylene tubes and stored at −80°C until further analysis.

The profiles of cytokines and inflammatory mediators were examined using a human cytokine antibody array (RayBiotech, USA) containing 80 cytokines and inflammatory mediators related to immune and inflammatory responses (**Supplementary Table 1**) according to the manufacturer’s instructions.

### Proteomic analysis in CSF samples of anti-NMDAR encephalitis patients and controls

Protein expression profile data were downloaded and processed by R and Bioconductor packages. Then, the “limma” package in R (version 3.6.3) was performed to screen for differentially expressed cytokines and inflammatory mediators. Adjusted *p*-value < 0.05 and ∣log2 Fold Change∣ > 1.2 were used as the cutoff criteria. In order to visualize differentially expressed cytokines and inflammatory mediators, volcanic maps and heat maps were drawn using the R package “ggplot2” and “pheatmap”.

### Pathway and functional analysis of differentially expressed cytokines and inflammatory mediators

The Gene Ontology (GO) website (http://www.geneontology.org/) was used to analyze the biological process (BP), the cellular component (CC), and the molecular function (MF) of differentially expressed cytokines and inflammatory mediators. The Kyoto Encyclopedia of Genes and Genomes (KEGG) website (http://www.genome.jp/kegg/) was used for pathway analysis to link protein information with higher-order functional information. The R package “clusterProfiler” was used to analyze and to identify biological states and pathway processes affected by differentially expressed cytokines and inflammatory mediators. We defined enriched functions and pathways using a cutoff of *p* < 0.05.

### Enzyme-linked immunosorbent assay

In cohort 2, commercially available ELISA kits were used to measure the concentrations of CXCL13 (Bioswamp, Wuhan, China), MCSF (Bioswamp, Wuhan, China), NFL (Bioswamp, Wuhan, China), NLRP3 (Bioswamp, Wuhan, China), NSE (Bioswamp, Wuhan, China), cIAP-1 (Ruixin Biotech Co., Ltd., Quanzhou, China), and LAMP-1 (Ruixin Biotech Co., Ltd., Quanzhou, China) in CSF according to the manufacturers’ instructions. The OD_450_ values were detected using the microplate reader (Allsheng, Hangzhou, China). A standard curve was created by reducing the data using computer software capable of performing four-parameter logistic curve fitting, and the best-fit line was determined by regression analysis.

### Statistical analysis

The Shapiro–Wilk test was used for normality test. A *p*-value of >0.05 was considered normal distribution. The measurement data that correspond to normal distribution were expressed in mean differences ± standard deviation. The other data that did not conform to a normal distribution were represented by median (maximum, minimum), and the enumeration data were expressed as case number (percentage). The one-way analysis of variance (ANOVA) with Bonferroni-corrected *post-hoc* tests was performed to compare the levels of cytokines and inflammatory mediators among the three groups. Pearson correlation analysis was used to analyze the data that conformed to a normal distribution, Spearman test was adopted for the correlation analysis of data with non-normal distribution, and point-biserial correlation was used for the dichotomic variables. To evaluate the performance of differentially expressed proteins in differentiating anti-NMDAR encephalitis from viral encephalitis and determine the threshold values, the receiver operating characteristic (ROC) analyses were used. Cutoff points for CSF differentially expressed proteins with diagnostic value were determined by likelihood ratio positive = sensitivity/(1 − specificity) from described ROC curves. All statistical analyses were performed using SPSS version 26.0 (IBM Corp, Armonk, NY, USA). A *p*-value of <0.05 was considered as significance level.

## Results

### Baseline characteristics of study subjects

A total of 19 patients with anti-NMDAR encephalitis, 19 controls, and 13 viral encephalitis patients were enrolled in this study. All patients had positive CSF and serum anti-NMDAR antibodies. Among them, 6 patients with anti-NMDAR encephalitis and 6 controls were included in cohort 1, and 13 patients in each of the three groups were included in cohort 2. The two cohorts were completely independent.

There was no significant difference in age and gender between these groups from both cohorts. In cohort 2, 7 patients showed headache, 4 cases had fever, 8 cases presented with psychiatric and behavioral symptoms, 10 cases presented with seizures, 3 cases had autonomic dysfunction, 3 cases had motor dysfunction, 3 cases had speech disorders, 4 cases had a decrease in level of consciousness, and 3 cases had central hypoventilation. Ovarian teratoma was found in two patients, aged 31 and 36, respectively, and both of them had their teratoma removed. Five patients had abnormal brain MRI, and lesions were mainly located in the temporal, hippocampus, parietal, and occipital lobes. After admission, all of these 13 anti-NMDAR encephalitis patients were treated with corticosteroids and intravenous immunoglobulin (IVIg). Other clinical data are shown in **Table 1**.

**Table 1:** Clinical characteristics of study population in cohort 2.

	NMDARE group	Control group	VE group
Demographics			
Gender (male/female)	6/7	5/8	8/5
Age (years)	30.54 ± 9.96	31.31 ± 11.58	33.23 ± 11.04
Clinical features			
Prodromic infection	5 (38.46%)	NA	NA
Duration of the disease (days)	27 (9, 144)	NA	NA
mRS in the acute phase	4 (1, 5)	NA	NA
mRS at 3-month follow-up	1 (0, 5)	NA	NA
NEOS score	1.54 ± 1.27	NA	NA
Abnormal brain MRI	5 (38.46%)	NA	NA
Abnormal EEG	7 (53.85%)	NA	NA
CSF finding			
Leukocytosis, *n* (%)	12 (92.31%)	NA	NA
Hyperproteinorrachia, *n* (%)	5 (38.46%)	NA	NA
Antibody titer in the CSF	1:100 (1:10, 1:100)	NA	NA

Data are presented as mean ± SD for normally distributed continuous variables, and as median (maximum, minimum) for non-normally distributed continuous. The enumeration data are n (%) or n/N.

NMDARE, anti-N-methyl-D-aspartate receptor encephalitis; VE, viral encephalitis; mRS, modified Rankin Scale; MRI, magnetic resonance imaging; NEOS, anti-NMDAR Encephalitis One-Year Functional Status; EEG, electroencephalography; CSF, cerebrospinal fluid; Leukocytosis > 5 cells/µl, Hyperproteinorrachia > 45 mg/dl.

NA, Not available.

### CSF differentially expressed cytokines and inflammatory mediators discovery in cohort 1

**Figure 1:**
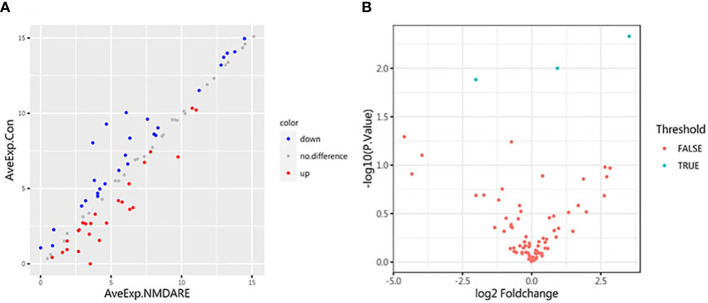
Scatter plot and volcano plot. **(A)** The abscissa and ordinate are the average expression of CSF cytokines in the anti-NMDAR encephalitis group and the control group, respectively. Red dots represent upregulated proteins. Blue dots represent downregulated proteins. **(B)** Volcano plot for identified proteins in CSF between anti-NMDAR encephalitis patients and control group patients. The differences in 80 cytokines between the anti-NMDAR encephalitis patients and the control group are shown according to fold change (*x*-axis, Log2 fold change) and *p*-value (*y*-axis, −log10 *p*-value). Blue dots represent cytokines or inflammatory mediators with *p*-value < 0.05 and fold change > 1.2 or < 0.83, from left to right: LAMP-1, MCSF, and cIAP-1; red dots denote cytokines or inflammatory mediators with *p*-value > 0.05, or 0.83 < fold change < 1.2.

**Figure 2:**
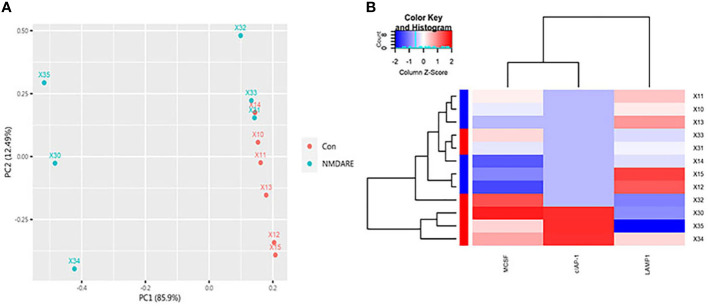
PCA and Hierarchical cluster analysis. **(A)** The array data of the differential proteins were analyzed *via* PCA. The two principal components are plotted to show the differences between the groups (control group in red and anti-NMDAR encephalitis group in blue). **(B)** Hierarchical cluster analysis exactly distinguished the anti-NMDAR encephalitis group from the control group. Each column is a protein, from left to right on the abscissa: MCSF, cIAP-1, and LAMP-1. Each row is a patient. Blue indicates low protein levels, white indicates median levels, and red denotes high levels.

In cohort 1, differentially expressed cytokines and inflammatory mediators were screened from 80 cytokines and inflammatory mediators associated with immune and inflammatory responses by cytokine array analysis. The Student’s *t*-test showed three cytokines and inflammatory mediators showed significantly different abundances between anti-NMDAR encephalitis patients and non-inflammatory diseases patients (*p* < 0.05) (**Table 2**). The scatter plot and volcano plot of differentially expressed cytokines and inflammatory mediators were used to visualize the magnitude of changes of the quantitative data (**Figure 1**). As shown in the volcano plot, among these three differentially expressed cytokines and inflammatory mediators, cIAP-1 and MCSF showed higher levels in CSF of anti-NMDAR encephalitis patients than of controls, while LAMP-1 had lower level in anti-NMDAR encephalitis patients. The principal component analysis (PCA) and hierarchical clustering were conducted on all differentially expressed cytokines and inflammatory mediators to show and analyze dissimilarities between two groups (**Figure 2**).

**Table 2:** Differentially expressed cytokines and inflammatory mediators in anti-NMDAR encephalitis in cohort 1.

Protein	AveExp.NMDARE	AveExp.Con	logFC	*p*-value	Adj. *p*-value	Fold change	Regulation
cIAP-1	3.5270	0	3.5270	0.0047	0.3488	11.5277	Up
LAMP-1	7.5879	9.6062	−2.0183	0.0131	0.3488	0.2468	Down
MCSF	1.8775	0.9484	0.9290	0.0100	0.3488	1.9040	Up

Table lists the three CSF biomarker candidates for NMDARE. A positive fold change indicates that the protein is upregulated in the NMDARE group in contrast to the control group. Differential analysis between groups was undertaken using linear modeling with the package LIMMA. A negative fold change indicates that the protein is downregulated in the NMDARE group compared to the control group. p-value: moderated t-statistics; Adjust p-value: Benjamini–Hochberg.

NMDARE, anti-N-methyl-D-aspartate receptor encephalitis; Con, controls; cIAP-1, Cellular inhibitor of apoptosis protein; LAMP-1, Lysosome-associated membrane protein 1; MCSF, macrophage colony-stimulating factor.

### GO term and KEGG pathway enrichment analysis

The analyses of GO enrichment and KEGG pathway enrichment were carried out to reveal the biological functions of differentially expressed cytokines and inflammatory mediators, and to clarify the possible pathogenesis of anti-NMDAR encephalitis. The results showed that there were 240 GO terms, of which 199 were BP terms, 32 were CC terms, and 9 were MF terms.

**Figure 3:**
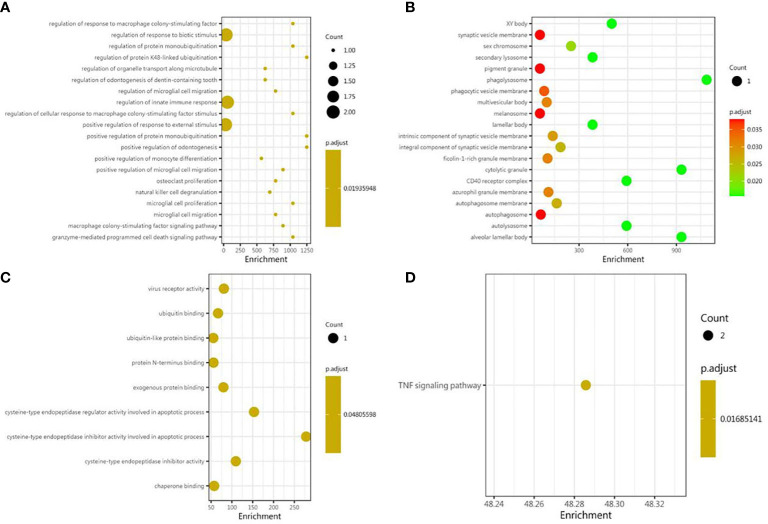
Bioinformatic analysis of differential proteins. Using Fisher’s accurate test, *p* < 0.05 was considered statistically significant. GO enrichment analysis included three subtypes: **(A)** cellular component; **(B)** molecular function; and **(C)** biological process. **(D)** The KEGG pathway enrichments.

As shown, in the GO BP category, differentially expressed cytokines and inflammatory mediators were mostly involved in the regulation of cytokine-mediated signaling pathway, the regulation of response to cytokine stimulus, the regulation of innate immune response, the positive regulation of protein monoubiquitination, the regulation of response to macrophage colony-stimulating factor, the regulation of cellular response to macrophage colony-stimulating factor stimulus, the regulation of microglial cell migration, the granzyme-mediated apoptotic signaling pathway, the macrophage colony-stimulating factor signaling pathway, and the regulation of protein monoubiquitination (**Figure 3A**). In the GO CC category, differentially expressed cytokines and inflammatory mediators were primarily enriched in phagolysosome, cytolytic granule, alveolar lamellar body, autolysosome, CD40 receptor complex, XY body, secondary lysosome, lamellar body, sex chromosome, and the integral component of synaptic vesicle membrane (**Figure 3B**). In the GO MF category, differentially expressed cytokines and inflammatory mediators were enriched in cysteine-type endopeptidase inhibitor activity involved in apoptotic process, cysteine-type endopeptidase regulator activity involved in apoptotic process, cysteine-type endopeptidase inhibitor activity, virus receptor activity, exogenous protein binding, ubiquitin binding, ubiquitin-like protein binding, chaperone binding, and protein N-terminus binding (**Figure 3C**). The results of KEGG pathway enrichment showed that the most involved pathway was the TNF signaling pathway (**Figure 3D**).

### Verification by ELISA in an expanded cohort 2

**Figure 4:**
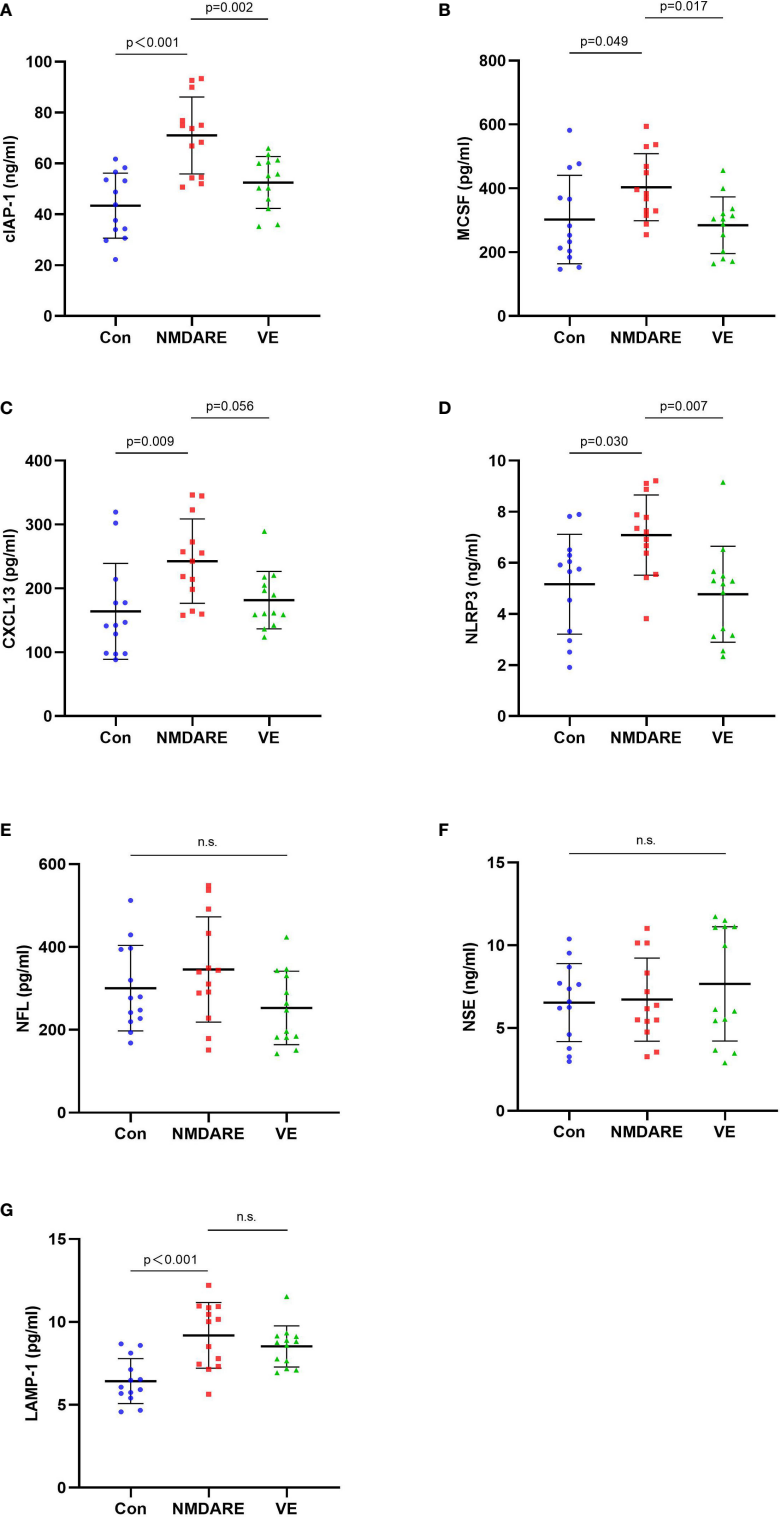
The CSF levels of cytokines and inflammatory mediators among the control group, anti-NMDAR encephalitis group, and viral encephalitis group in the validation cohort detected by ELISA. **(A)** Cellular inhibitor of apoptosis protein (cIAP-1) in CSF among the three groups. **(B)** Macrophage colony-stimulating factor (MCSF) in CSF among the three groups. **(C)** CXC chemokine ligand 13 (CXCL13) in CSF among the three groups. **(D)** Nucleotide binding oligomerization domain-like receptor protein 3 (NLRP3) in CSF among the three groups. **(E)** Neurofilament light chain (NFL) in CSF among the three groups. **(F)** Neuron-specific enolase (NSE) in CSF among the three groups. ELISA, enzyme-linked immunosorbent assay. Data are expressed as mean ± SD. *p*-value: One-way ANOVA. *n* = 13/group. n.s. indicates no significant differences among the groups. **(G)** Lysosome-associated membrane protein 1 (LAMP-1) in CSF among the three groups.

In order to validate the differentially expressed cytokines and inflammatory mediators identified by cytokine array analysis and some other candidate biomarkers reported in previous literatures, ELISA was carried out to verify cohort 2. The results showed that the expressions of cIAP-1, MCSF, CXCL13, and NLRP3 were higher in the anti-NMDAR encephalitis group than controls and the viral encephalitis group (*p* < 0.05) (**Table 3** and **Figure 4**). The LAMP-1 in CSF of anti-NMDAR encephalitis patients was higher than that of the control group, but that from the anti-NMDAR encephalitis group and the viral encephalitis group was not statistically different.

**Table 3:** Comparison of CSF seven cytokines and inflammatory mediator levels among anti-NMDAR encephalitis, controls, and viral encephalitis.

	NMDARE(*n* = 13)	Con(*n* = 13)	VE(*n* = 13)	*F* value	*p*-value
cIAP-1 (ng/ml)	70.12 ± 15.55	43.39 ± 12.79	52.48 ± 10.19	15.553	<0.001
MCSF (pg/ml)	404.65 ± 102.07	301.99 ± 138.58	284.05 ± 88.74	4.230	0.022
LAMP-1 (ng/ml)	9.57 ± 1.78	6.43 ± 1.36	8.52 ± 1.24	11.075	<0.001
CXCL13 (pg/ml)	238.28 ± 67.45	163.91 ± 75.05	181.52 ± 44.84	5.544	0.008
NLRP3 (ng/ml)	6.96 ± 1.52	5.16 ± 1.95	4.77 ± 1.88	6.121	0.005
NFL (pg/ml)	349.64 ± 128.67	300.67 ± 103.25	252.77 ± 88.62	2.428	0.103
NSE (ng/ml)	6.79 ± 2.22	6.54 ± 2.35	7.67 ± 3.46	0.607	0.550

Data are presented as mean ± SD. p-value: one-way ANOVA.

NMDARE, anti-N-methyl-D-aspartate receptor encephalitis; CSF, cerebrospinal fluid; Con, controls; cIAP-1, Cellular inhibitor of apoptosis protein; MCSF, macrophage colony-stimulating factor; LAMP-1, Lysosome-associated membrane protein 1; CXCL13, CXC chemokine ligand 13; NLRP3, nucleotide binding oligomerization domain-like receptor protein 3; NFL, neurofilament light chain; NSE, neuron-specific enolase.

### Correlation between differentially expressed cytokines or inflammatory mediators and critical clinical features

**Figure 5:**
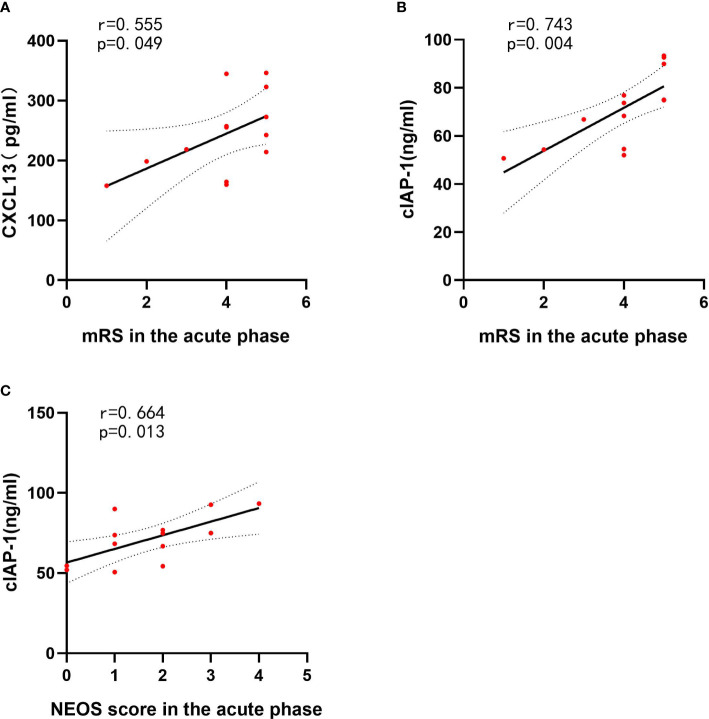
CSF cytokines and inflammatory mediator levels correlated with clinical features of anti-NMDAR encephalitis patients. The *p*-values represent the results of the Spearman’s rank correlation coefficient or point-biserial correlation coefficient. *p* < 0.05. CSF, cerebrospinal fluid; NMDAR, anti-N-methyl-D-aspartate receptor; mRS, modified Rankin Scale; NEOS, anti-NMDAR Encephalitis One-Year Functional Status; CXCL13, CXC chemokine ligand 13; MCSF, macrophage colony-stimulating factor; NLRP3, nucleotide binding oligomerization domain-like receptor protein 3. **(A)** The correlations between CXCL13 and mRS in the acute phase of anti-NMDAR encephalitis patients. **(B)** The correlations between cIAP-1 and mRS in the acute phase of anti-NMDAR encephalitis patients. **(C)** The correlations between cIAP-1 and NEOS score in the acute phase of anti-NMDAR encephalitis patients.

To assess the association between clinical features and differentially expressed protein levels of anti-NMDAR encephalitis patients, linear correlation analysis was performed. Linear regression revealed a correlation between high CSF levels of CXCL13 and the highest mRS score in the acute phase (*r* = 0.555, *p* = 0.049). The CSF cIAP-1 level was also positively correlated with the highest mRS score in the acute phase and the NEOS score (*r* = 0.743, *p* = 0.004; *r* = 0.664, *p* = 0.013) (**Figure 5**). However, no associations between the levels of differentially expressed proteins and other clinical features were identified, where the detailed information is shown in **Table 4**.

**Table 4:** CSF cytokines and inflammatory mediator levels correlated with clinical features of anti-NMDAR encephalitis patients.

Cytokines or inflammatory mediators	Gender		Age		Duration of the disease		Prodromic infection	
	*r*	*p*	*r*	*p*	*r*	*p*	*r*	*p*
cIAP-1 (ng/ml)	0.311	0.301	0.100	0.745	0.436	0.137	0.125	0.685
MCSF (pg/ml)	−0.367	0.217	0.252	0.407	−0.029	0.926	−0.220	0.469
CXCL13 (pg/ml)	−0.160	0.602	−0.187	0.540	0.195	0.522	−0.325	0.279
NLRP3 (ng/ml)	0.180	0.555	−0.125	0.684	0.199	0.515	−0.428	0.144
Cytokines or inflammatory mediators	mRS in the acute phase		mRS at 3-month follow-up		NEOS score		Protein level in CSF	
	*r*	*p*	*r*	*p*	*r*	*p*	*r*	*p*
cIAP-1 (ng/ml)	0.743	0.004*	0.467	0.108	0.727	0.005*	0.257	0.398
MCSF (pg/ml)	−0.135	0.659	−0.172	0.575	−0.083	0.788	0.200	0.512
CXCL13 (pg/ml)	0.555	0.049*	0.323	0.281	0.076	0.806	0.023	0.940
NLRP3 (ng/ml)	−0.225	0.459	−0.011	0.971	−0.161	0.598	−0.247	0.417
Cytokines or inflammatory mediators	WBC counts in CSF		Antibody titer in the CSF		Abnormal EEG		Abnormal brain MRI	
	*r*	*p*	*r*	*p*	*r*	*p*	*r*	*p*
cIAP-1 (ng/ml)	0.418	0.155	0.331	0.269	0.075	0.808	0.203	0.506
MCSF (pg/ml)	−0.202	0.509	−0.245	0.421	−0.235	0.440	−0.163	0.595
CXCL13 (pg/ml)	−0.104	0.734	0.069	0.824	−0.055	0.859	−0.043	0.889
NLRP3 (ng/ml)	−0.091	0.768	−0.305	0.311	0.162	0.596	−0.139	0.650

The p-values represent the results of Spearman’s rank correlation coefficient or point-biserial correlation coefficient. *p < 0.05.

NMDAR, anti-N-methyl-D-aspartate receptor; mRS, modified Rankin Scale; MRI, magnetic resonance imaging; NEOS, anti-NMDAR Encephalitis One-Year Functional Status; EEG, electroencephalography; CSF, cerebrospinal fluid; WBC, white blood cell count; cIAP-1, Cellular inhibitor of apoptosis protein; MCSF, macrophage colony-stimulating factor; CXCL13, CXC chemokine ligand 13; NLRP3, nucleotide binding oligomerization domain-like receptor protein 3.

### ROC curve of the differentially expressed proteins

To analyze the abilities and accuracy of differentially expressed proteins in CSF to distinguish anti-NMDAR encephalitis from viral encephalitis, the AUC and their 95% CIs were used from ROC analyses (**Supplementary Figure**). The area under the ROC curve (AUC) was calculated to indicate efficiency and accuracy of identification. The results showed that the AUCs of cIAP-1, MCSF, CXCL13, and NLRP3 were 0.834, 0.808, 0.781, and 0.852, respectively; the sensitivities were 1, 0.692, 0.846, and 0.692, respectively; and the specificities were 0.846, 0.846, 0.615, and 0.923 respectively. The cutoff values of four potential biomarkers of cIAP-1, MCSF, CXCL13, and NLRP3 in diagnosing anti-NMDAR encephalitis were 66.37 ng/ml, 314.5 pg/ml, 218.1 pg/ml, and 5.36 ng/ml respectively. The results suggested that these four biomarkers exhibited potential values in predicting anti-NMDAR encephalitis, and detailed parameters are displayed in **Supplementary Table 2**. Based on the above data, these four differentially expressed proteins in CSF may serve as potential biomarkers for differentiation of anti-NMDAR encephalitis from viral encephalitis.

## Discussion

Recent studies have suggested the increased expressions of some inflammatory proteins in CSF of anti-NMDAR encephalitis patients, which might play crucial roles in the evaluation of disease severity and prediction of prognosis. However, unclear regulation mechanism and prognostic value of inflammatory proteins and their associations with clinical factors in anti-NMDAR encephalitis are available owing to the limited number of studies on inflammatory proteins and the lack of large-scale screening studies.

Here, we opted for a quantibody human inflammation array that could simultaneously probe patients’ CSF samples for 80 cytokines and inflammatory mediators related to immune and inflammatory responses. We recognized three differentially expressed cytokines and inflammatory mediators (containing two upregulated cytokines and one downregulated and inflammatory mediator) and then performed protein function analysis. As shown, the differentially expressed cytokines and inflammatory mediators were mostly involved in inflammatory responses (such as regulation of innate immune response, positive regulation of microglial cell migration, and regulation of response to MCSF) and homeostasis regulation (including regulation of phagocytosis, autophagy, protein ubiquitination, and apoptotic process). The differentially expressed cytokines and inflammatory mediators mainly participated in the TNF signaling pathway according to KEGG. These findings suggest the involvement of multiple pathways during anti-NMDAR encephalitis development, onset of symptoms, or disease progression, and may also provide insights into pathological processes involved in anti-NMDAR encephalitis. After ELISA validation, we screened and positively validated four promising CSF biomarker candidates for anti-NMDAR encephalitis, namely, cIAP-1, MCSF, CXCL13, and NLRP3. Furthermore, all these biomarker candidates were upregulated in anti-NMDAR encephalitis, and they had favorable diagnostic values. We will discuss these biomarker candidates below.

As a member of the ubiquitously expressed ubiquitin ligase family, cIAP-1 can inhibit cell apoptosis, regulate immunity, and maintain cell homeostasis. As an important regulator of the apoptosis cascade processes, cIAP-1 can block endogenous and exogenous cell apoptosis by directly binding and inhibiting activated different cysteinyl aspartate-specific proteinase (caspase) (13–15). It can also play an important role in the processes of congenital cellular immunity and acquired cellular immunity (16). Upregulated cIAP-1 can promote endothelial cell activation and leukocyte recruitment, and further participate in the inflammatory response process (17, 18). Furthermore, cIAP-1 can participate in regulating autophagy and maintaining homeostasis, and an elevated level of cIAP-1 could further induce the formation of autophagosomes and activate autophagy (19, 20). Under the conditions of starvation stress, ubiquitinated cIAP-1 could promote the mitochondrial autophagy process, leading to mitochondrial dysfunction and ATP production decline (21). We detected increased CSF cIAP-1 concentrations in anti-NMDAR encephalitis patients compared to controls, and the ROC curve analysis confirmed the dependability of its diagnostic value. Since there have been few research reports, the relationship between cIAP-1 and anti-NMDAR encephalitis might be an interesting finding. Higher abundance of cIAP-1 may reflect potential pathophysiological processes in anti-NMDAR encephalitis, which may be related to the inhibition of apoptosis, activation of autophagy, and regulation of immune responses.

MCSF is a key regulator of the monocyte/macrophage system. It is widely expressed in various cells and tissues, and plays important roles in regulating the survival, proliferation, and differentiation of macrophages. The expression levels are low in normal physiological conditions, but it can increase rapidly in inflammatory conditions (22, 23). In the CNS, MCSF can exert anti-inflammatory effects by promoting microglia to an anti-inflammatory phenotype and regulating their phagocytic activities, while promoting anti-inflammatory factor release and tissue repair (24, 25). Myelin integrity can also be protected by MCSF-induced survival and proliferation of oligodendrocytes (26). We detected significant elevated MCSF in CSF from anti-NMDAR encephalitis individuals, which may reflect that microglial polarization and neuroinflammation may play a role in the pathogenesis of anti-NMDAR encephalitis. However, its specific mechanism should be further investigated.

LAMP-1 plays an important role in maintaining lysosomal stability, regulating immune response and the inflammatory process. LAMP-1 is a highly glycosylated protein with rich content in lysosomal membrane and is considered to be a marker of lysosomal stability (27). Studies have shown that increased expression of LAMP-1 could suggest enhanced cellular phagocytic activity and activated autolysosomal pathway (28–30), whereas the deficiency of LAMP-1 can lead to autophagosome accumulation and dysfunction in patients, which can be seen in various metabolic, neurodegenerative, and infectious diseases (31). In addition, LAMP-1 is also considered to be critical for the activations of NK cells and CD8+ T cells, playing an important role in NK cells exerting cytotoxicity as well as delivery of perforin to target cells (32). In CNS, LAMP-1 is mainly expressed on the surface of neurons and microglia, and may be partially involved in the neuroinflammatory process. In our study, cytokine array analysis results showed the downregulation of LAMP-1 levels in the CSF of patients with anti-NMDAR encephalitis. However, the result of ELISA was opposite and there was no significant difference between the anti-NMDAR encephalitis and viral encephalitis groups detected. Given few reports of its expression at this disease, the mechanism of LAMP-1 in anti-NMDAR encephalitis remains unclear and it needs to be paid more attention in future research.

CXCL13 is a member of the CXC chemokine family and it is mainly distributed in the cytoplasm and cell membrane of astrocytes and neurons (33). It can be involved in B-cell transport and CD4 ^+^ T-cell activation, stimulate antibody production, and further participate in immune and inflammatory responses. In addition, CXCL13 can regulate proliferation, differentiation, and apoptosis of hippocampal neural stem cells (34, 35). CXCL13, a vital immune inflammatory response factor, was reported to be involved in the occurrences of CNS diseases such as multiple sclerosis (MS), primary CNS lymphoma, epilepsy, cerebrospinal meningitis, and meningitis (36, 37). Evidence has suggested that in the acute phase of anti-NMDAR encephalitis, the content of CXCL13 in CSF increased significantly, and was related to older age, presence of infection symptoms before onset, combined with teratoma, and poor immunotherapy effect. The CXCL13 in the patient’s CSF can also increase again upon disease recurrence, suggesting that CXCL13 may be an indicator of anti-NMDAR encephalitis activity (5). In line with some of these studies, we found that the levels of CXCL13 in CSF of anti-NMDAR encephalitis patients were notably increased compared with controls. However, the specific mechanism needs further investigation in order to clarify whether CXCL13 participates in the development of anti-NMDAR encephalitis by regulating B cell- and T cell-mediated immune responses.

NLRP3 is an important component of the body’s innate immunity, which can activate the downstream inflammatory cytokines to trigger the inflammatory response after being activated by exogenous signals (various microorganisms and their metabolites) and endogenous signals (metabolites *in vivo*) (38). Activated NLRP3 can inhibit autophagy in microglia, further causing neurodegeneration. It has been shown that NLRP3 is elevated in patients with Alzheimer’s disease (39), viral encephalitis (40), MS (41), traumatic brain injury (42), stroke (43), Parkinson’s disease (44), epilepsy (45), and other diseases. Previous studies have shown that NLRP3 is significantly higher in the CSF of patients with anti-NMDAR encephalitis, and is associated with poor prognosis (6). Consistent with previous studies, our results also showed that NLRP3 was elevated in the CSF of patients with anti-NMDAR encephalitis, and its mediated inflammatory process may play an important role in the pathogenesis of anti-NMDAR encephalitis.

As a major component of the neuronal cytoskeleton, NF is mainly found in axons. NFL, as one of the main components of NF, plays a vital role in the maintenance of axon caliber and structure and in the preservation of synaptic structure. The absence of NFL can affect axonal function (46). NFL is the most abundant as well as soluble subunit of NF and is regarded as the most reliable measurable specific biomarker of axonal damage and neuronal death (9). CSF NFL levels, reflecting neuroaxonal damage, are currently used as biomarkers of response to therapy and prognosis in several inflammatory diseases, neurodegenerative diseases, traumatic brain injury, cerebrovascular disease, and MS (47). Its amount of release depends on the severity of axonal damage (48). A study of 24 anti-NMDAR encephalitis patients and 21 controls showed, *via* ELISA, that in the acute phase of anti-NMDAR encephalitis, the content of NFL in CSF is significantly increased, and decreased with the improvement of the condition. The decrease in NFL after treatment was associated with an improvement in mRS scores of patients (49). However, our study suggested that compared with the controls, the levels of NFL in CSF of anti-NMDAR encephalitis patients increased, but it was not statistically significant. The neuronal damage during the development of anti-NMDAR encephalitis may still need to be further explored.

NSE is a glycolytic enzyme, which is seen as a marker of neuronal maturation due to its production at a later stage of neuronal differentiation (50). In various pathological conditions, such as ischemia, hypoxia, and inflammation, the NSE can enter through the disrupted neuronal cell membrane into the CSF and peripheral circulation. Levels of NSE in serum and CSF are correlated with the degree of neuronal damage, and are thus considered as sensitive biomarkers of neuronal damage (51). Serum NSE concentrations have been found to increase in diseases such as stroke, traumatic brain injury, MS, and Alzheimer’s disease (52). It has also been reported in studies that in the early stage of anti-NMDAR encephalitis, the elevation level of NSE in CSF is significant. CSF NSE level is associated with severe condition and poor prognosis, but not with the anti-NMDAR antibody titer. These results suggest that NSE content in CSF in the early stage may serve as a biomarker of prognosis of anti-NMDAR encephalitis (10). However, in our study, the expression of NSE in CSF was not significantly different between the anti-NMDAR encephalitis and control groups, which may need to be repeatedly validated in a larger queue.

To clarify the specific value of differentially expressed proteins in anti-NMDAR encephalitis, we also compared the expression of differentially expressed proteins in the anti-NMDAR encephalitis group and the viral encephalitis group. The results showed that cIAP-1, MCSF, LAMP-1, CXCL13, and NLRP3 were elevated in the CSF of patients with anti-NMDAR encephalitis. The ROC results suggest that differentially expressed proteins may be used as potential biomarkers for differentiating anti-NMDAR encephalitis and viral encephalitis. However, there was no significant difference in the levels of NFL, NSE, and LAMP-1 in CSF between anti-NMDAR encephalitis and viral encephalitis. Whether they participate in the pathogenesis of the two diseases needs to be confirmed by more studies. In summary, our test results suggest that cIAP-1, MCSF, CXCL13, and NLRP3 were elevated in the CSF of patients with anti-NMDAR encephalitis. Although the decrease of LAMP-1 was not proved by ELISA, this may be due to the fact that patients in the two cohorts were not exactly in the same disease period when CSF was collected. Combined with the results of GO function and KEGG pathway enrichment analysis, it suggested that these cytokines and inflammatory mediators may be involved in the occurrence and development of anti-NMDAR encephalitis by participating in the regulation of altered autophagy levels, microglial cell polarization, and cell death.

Autophagy is an important process to maintain body homeostasis, and both impaired or excessive activation of cellular autophagy can lead (53, 54). Kallergi et al. demonstrated that activation of NMDAR resulting in elevated levels of autophagy in the mice postsynaptic dendrite by inducing long-term depression is involved in the NMDAR-mediated excitotoxic effects (55). However, Yang et al. found that in a chronic brain hypoperfusion model, hypertonic saline upregulated the expression of NMDAR-2B subunit and autophagy in rat hippocampus, further improved synaptic plasticity, and subsequently alleviated spatial learning and memory impairment (56). Our study showed that the expression of cIAP-1 was upregulated in CSF of anti-NMDAR encephalitis patients, and the level of LAMP-1 was significantly different from that of the control group. These cytokines and inflammatory mediators were mainly involved in the regulation of autophagy level, suggesting that the autophagy process may play a certain role in the development of anti-NMDAR encephalitis, but its specific role and mechanism still needed to be further clarified.

Microglia are widely distributed in CNS and are the major cells exerting immune functions. In physiological circumstances, microglia mainly play a monitoring role and participate in maintaining internal environmental stability. However, in the case of minor pathological changes in CNS, they can be immediately activated and polarized to two phenotypes, M1 or M2 type, at different stages of different diseases. M1-type microglia have obvious neurotoxic effects, participating in neuroinflammatory responses, neuronal damage, and apoptosis by secretion of various proinflammatory cytokines and chemokines; M2-type microglia mainly participate in phagocytosis, including phagocyte debris and clearance of pathogens. It can also promote tissue repair by secreting anti-inflammatory factors and growth factors. It has been reported that in anti-NMDAR encephalitis, microglia can be recruited by the Toll-like receptor (TLR) system, polarize to M1 type, release IL-1β, further cause neuroinflammation, and thus lead to epilepsy (57). The autopsy results of a patient with refractory autoimmune encephalitis also showed T cells and significant microglia infiltration in the hippocampus and medial temporal lobes (58). Our study found that the changes of MCSF and LAMP-1 expression in the CSF of anti-NMDAR encephalitis patients may be related to the microglial functions, and that microglial activation and dynamic phenotypic alteration may be involved in the pathogenesis of anti-NMDAR encephalitis.

Neuronal loss is one of the important mechanisms of neuronal damage and organ dysfunction. Because the pathogenesis of the disease is not completely clear, it is still controversial whether there is cell loss in the early-stage anti-NMDAR encephalitis. Wright et al. found that there was no cell loss in the region of NMDAR and antibody binding (59). Moreover, Hughes et al. indicated that anti-NMDA receptor encephalitis can be reversible after early antibody removal (2). Rosenfeld et al. presented that the symptoms of anti-NMDAR encephalitis patients can be rapidly reversible if promptly diagnosed and treated (60). However, the autopsy results of a patient with anti-NMDAR encephalitis showed neuronal loss in the limbic system (61). The above studies suggest that the role of neuronal death in the pathogenesis of anti-NMDAR encephalitis is not clear. In this study, cIAP-1 was upregulated in CSF of anti-NMDAR encephalitis patients, and it can mediate the inhibition of apoptosis. The level of LAMP-1 in the CSF of patients with anti-NMDAR encephalitis was also significantly different from that of the controls, and it has been found to be involved in the autophagy process. However, their specific roles in this disease need further investigation. NFL and NSE were correlated with the neuronal damage to some extent. However, we did not find significant difference of their levels between two groups. In addition, KEGG analysis in this study showed that differentially expressed cytokines and inflammatory mediators were mainly involved in the TNF signaling pathway, which is crucial in TNF-induced cell death and is important target for the treatment of autoimmune diseases. Therefore, we consider that neuronal cell death in anti-NMDA receptor encephalitis can be used as a meaningful research target.

To further clarify the clinical value of differentially expressed proteins, their correlation with clinical features of patients with anti-NMDAR encephalitis was analyzed in this study. We collected clinical data of patients with anti-NMDAR encephalitis, including age, gender, situation of the combined precursor infection, duration of duration, maximum mRS score in the acute phase, mRS score at 3 months’ follow-up, NEOS score, CSF leukocyte count and protein levels, CSF antibody titer, and brain MRI and EEG, and then correlation analysis was performed between these data and cIAP-1, MCSF, CXCL13, and NLRP3 levels in CSF. The results showed that CXCL13 level was positively correlated with disease duration, highest mRS, mRS score at follow-up, and NEOS score; MCSF level was positively associated with the duration of disease; and NLRP3 level was obviously correlated with CSF protein content. However, there was no evident correlation between these levels of the inflammatory proteins and age, gender, presence of prodromal infection, CSF leukocyte count, anti-NMDAR antibody titer, and abnormal brain MRI and EEG in anti-NMDAR encephalitis patients. This suggests that, to some extent, CXCL13 content in CSF can be used as a biomarker to reflect disease severity and judge prognosis. Consistent with previous studies, the findings of these candidate biomarkers highlight the importance of neuroinflammatory and immune responses in anti-NMDAR encephalitis, and these inflammatory proteins may have comprehensive effects on pathological mechanisms in anti-NMDAR encephalitis by influencing different biological signaling pathways.

Several considerations and limitations should be noted in our study. The patients with anti-NMDAR encephalitis are from a single center with a small number of cases, which affects the test effectiveness of comparison between groups. Larger studies are still needed to further confirm the pathological mechanism of the above possible biomarkers in anti-NMDAR encephalitis. Furthermore, longer follow-up and CSF collection should also be performed to further evaluate inflammatory protein expressions at different stages of anti-NMDAR encephalitis and verify the relationship between inflammatory proteins levels and prognosis.

## Conclusion

In conclusion, our findings provide reference for the use of high-throughput technologies, especially cytokine array analysis, for identification of candidate anti-NMDAR encephalitis biomarkers. Furthermore, we have identified and positively validated CSF cIAP-1, MCSF, CXCL13, and NLRP3 as novel biomarker candidates, and their levels may reflect severity and predict prognosis of anti-NMDAR encephalitis. Considering that the decline of CSF LAMP-1 was not validated by ELISA in the second cohort, it remains to be determined whether significantly different concentrations would be found in larger cohorts. In addition, whether these candidate biomarkers can help clinically identify anti-NMDAR encephalitis and viral encephalitis also needs more attention in the future. These intriguing biomarker candidates should be explored in future prospective validation studies. Identification of these candidate biomarkers also strengthens the importance of microglial-mediated immune responses, altered levels of autophagy, and the TNF signal pathway in the pathophysiology of anti-NMDAR encephalitis, which warrant further research to clarify the occurrence and development process of the disease and to find potential new therapeutic targets.

## Data availability statement

The datasets used and/or analyzed during the current study are available from the corresponding author on reasonable request.

## Ethics statement

The study was performed according to the ethical principles of the Declaration of Helsinki and was approved by Ethics Committee of the first hospital of Shanxi Medical University. The patients provided their written informed consent to participate in this study.

## Author contributions

KY and YL put forward the concept and designed the study. FZ, HS and ML performed clinical data and CSF sample collection. KY and YL carried out experiments. JieW and YL analyzed the data. YL and FZ wrote the manuscript. JG and JieW reviewed the manuscript and finalized the paper. All authors contributed to the article and approved the submitted version.

## Funding

This work was supported by grants from “136” medical engineering Fund for scientific research, First Hospital of Shanxi Medical University.

## Acknowledgments

We thank all patients for their data and CSF donation and all medical staff at the Department of Neurology, the First Hospital of Shanxi Medical University for their help during the study.

## Conflict of interest

The authors declare that the research was conducted in the absence of any commercial or financial relationships that could be construed as a potential conflict of interest.

## Publisher’s note

All claims expressed in this article are solely those of the authors and do not necessarily represent those of their affiliated organizations, or those of the publisher, the editors and the reviewers. Any product that may be evaluated in this article, or claim that may be made by its manufacturer, is not guaranteed or endorsed by the publisher.

## Supplementary material

ROC curves of potential biomarkers for differentiating anti-NMDAR encephalitis and viral encephalitis. ROC curves showing relative expression levels of cIAP-1, MCSF, CXCL13 and NLRP3 used to differentiate between anti-NMDAR encephalitis patients and viral encephalitis patients. Abbreviations: NLRP3, nucleotide binding oligomerization domain—like receptor protein 3; CXCL13, CXC chemokine ligand 13; MCSF, macrophage colony-stimulating factor; cIAP-1, Cellular inhibitor of apoptosis protein.
