# Supplementary figures and images for: Identification of cerebrospinal fluid biomarker candidates for anti-N-methyl-D-aspartate receptor encephalitis: High-throughput proteomic investigation
Source: Front Immunol. 2022 Oct 26;13:971659. doi: 10.3389/fimmu.2022.971659 (PMC9643472; doi:10.3389/fimmu.2022.971659)

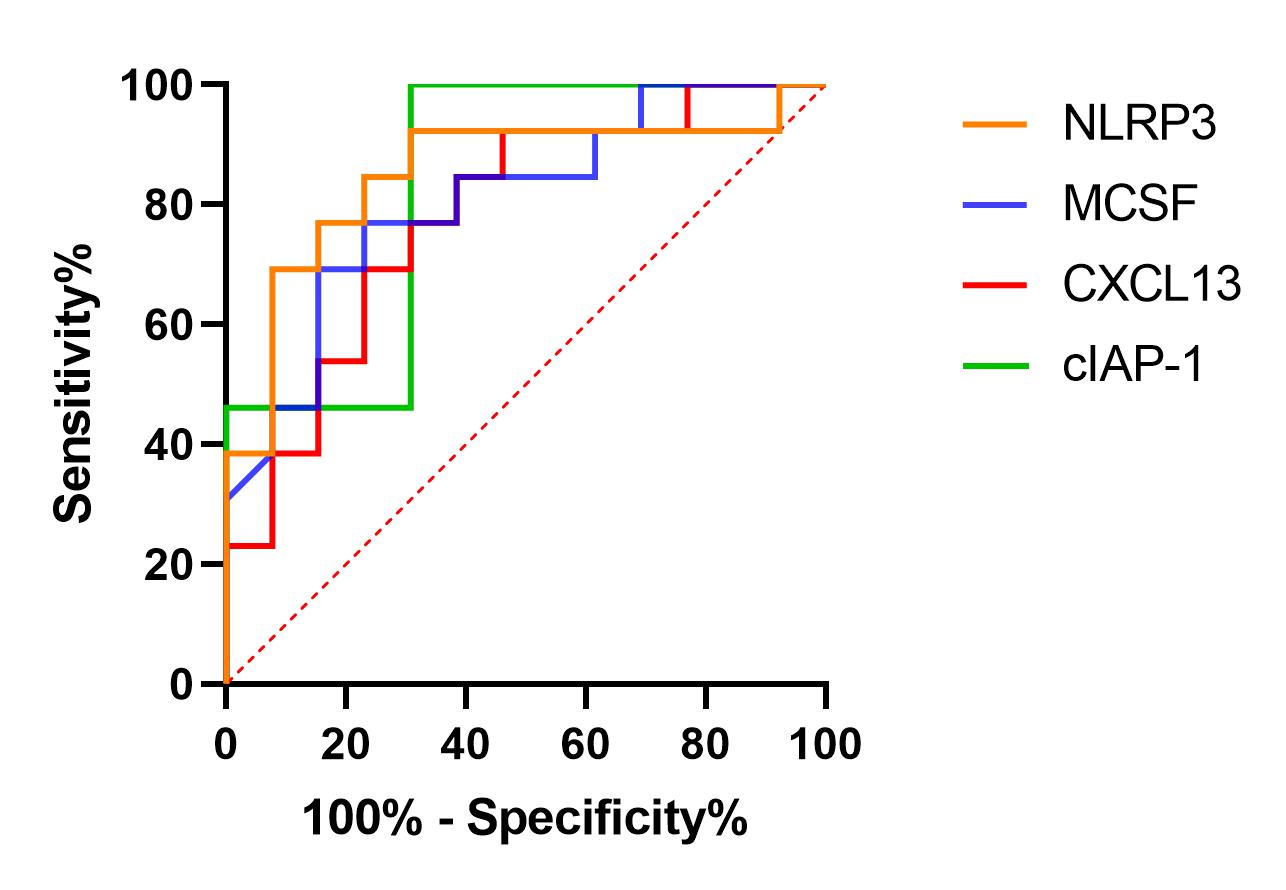

Supplement: Supplementary file 1 [file Image_1.jpg]
