# Supplementary material for: Identification of cerebrospinal fluid biomarker candidates for anti-N-methyl-D-aspartate receptor encephalitis: High-throughput proteomic investigation
Source: Front Immunol. 2022 Oct 26;13:971659. doi: 10.3389/fimmu.2022.971659 (PMC9643472; doi:10.3389/fimmu.2022.971659)
Supplement: Supplementary file 2 [file Table_1.docx]

**Supplementary table 1. Eighty cytokines and inflammatory mediators associated with immune and inflammatory responses of cytokine array analysis**

| Protein ID | | Full name | | | Entrez ID | | Uniport ID |
| --- | --- | --- | --- | --- | --- | --- | --- |
| cIAP-1 | | Cellular inhibitor of apoptosis protein-1 | | | 329 | | Q13490 |
| MCSF | | Macrophage colony-stimulating factor | | | 3916 | | P11279 |
| LAMP-1 | | Lysosome-associated membrane protein-1 | | | 1435 | | P09603 |
| LGMN | | Legumain | | | 5641 | | Q99538 |
| ICAM-3 | | intercellular adhesion molecule -3 | | | 3385 | | P32942 |
| MSP-R | | macrophage stimulating protein receptor | | | 4486 | | Q04912 |
| SH2D1A | | SH2 domain protein 1A | | | 4068 | | 060880 |
| XIAP | | X-linked inhibitor of apoptosis | | | 331 | | P98170 |
| I-309/CCL1 | | Chemokine ligand 1 | | | 6346 | | P22362 |
| IL-12p70 | | Interleukin-12p70 | | | 3592 | | P29459 & P29460 |
| MDC | | macrophage-derived chemokine | | | 6367 | | 000626 |
| TLR4 | | Toll like receptor 4 | | | 7099 | | 000206 |
| CD137 | | cluster of differentiation 137 | | | 8744 | | P41273 |
| 6Ckine/SLC | | Secondary lymphoid tissue chemokine | | | 6366 | | 000585 |
| ADAM8 | | a disintegrin and metalloproteinase domain-containing protein 8 | | | 101 | | P78325 |
| Bcl-10 | | B-cell lymphoma-10 | | | 8915 | | 095999 |
| CALR | | Calreticulin | | | 811 | | P27797 |
| Caspase 8 | | | cysteinyl aspartate specific proteinase 8 | 841 | | | Q14790 |
| CTSL | | | Cathepsin L | 1514 | | | P07711 |
| CTSS | | | Cathepsin S | 1520 | | | P25774 |
| CD84 | | | cluster of differentiation 84 | 8832 | | | Q9UIB8 |
| cIAP-2 | | | Cellular inhibitor of apoptosis protein-2 | 330 | | | Q13489 |
| CLEC10A | | | C-type lectin-like receptor 10A | 10462 | | | Q8IUN9 |
| CLU | | | Clusterin | 1191 | | | P10909 |
| CR-1 | | | Cripto-1 | 6997 | | | P13385 |
| Dectin-1 | | | dendritic cell-associated C-type lectin-1 | 64581 | | | Q9BXN2 |
| EDAR | | | ectodysplasin A receptor | 60401 | | | Q9HAV5 |
| FCRL1 | | | Fc receptor-like 1 | 115350 | | | Q96LA6 |
| Gal-3 | | | Galectin-3 | 3958 | | | P17931 |
| Gal-9 | | | Galectin-9 | 3965 | | | 000182 |
| Gas6 | | | Growth Arrest Specific Protein 6 | 2621 | | | Q14393 |
| GITR L | | | glucocorticoid induced TNF receptor ligand | 8995 | | | Q9UNG2 |
| IFNb | | | Interferon b | 3456 | | | P01574 |
| IFNg | | | Interferon g | 3458 | | | P01579 |
| IL-15 | | | Interleukin-15 | 3600 | | | P40933 |
| IL-21 | | | Interleukin-21 | 59067 | | | Q9HBE4 |
| IL-23 | | | Interleukin-23 | 51561 | | | Q9NPF7 |
| IL-23 R | | | Interleukin-23 receptor | 149233 | | | Q5VWK5 |
| integrin α-M | | | integrin subunit alpha-M | 3684 | | | P11215 |
| MDL-1 | | | Myeloid DAP12-associating lectin-1 | 23601 | | | Q9NY25 |
| MMP-2 | | | Matrix metalloproteinase-2 | 4313 | | | P08253 |
| NKp44 /NCR2 | | | natural cytotoxicity triggering receptor 2 | 9436 | | | 095944 |
| NKp46/NCR1 | | | natural cytotoxicity triggering receptor 1 | 9437 | | | 076036 |
| PU.1 | | | Transcription factor PU.1 | 6688 | | | P17947 |
| TC-PTP | | | T Cell Protein Tyrosine Phosphatase | 5771 | | | P17706 |
| TREM-2 | | | Triggering receptor expressed on myeloid cells 2 | 54209 | | | Q9NZC2 |
| Axl | | | anexelekto | 558 | | | P30530 |
| CTSB | | | Cathepsin B | 1508 | | | P07858 |
| CD14 | | | cluster of differentiation 14 | 929 | | | P08571 |
| CRTAM | | | Class-I Restricted T Cell-Associated Molecule | 56253 | | | 095727 |
| CXCL16 | | | CXC chemokine ligand 16 | 58191 | | | Q9H2A7 |
| Eotaxin-2 | | | eosinophil chemotactic protein-2 | 6369 | | | 000175 |
| Eotaxin-3 | | | eosinophil chemotactic protein-1 | 10344 | | | Q9Y258 |
| FKN | | | Fractalkine | 6376 | | | P78423 |
| ICAM-1 | | | intercellular adhesion molecule-1 | 3383 | | | P05362 |
| IGF-2 | | | insulin-like growth factor-2 | 3481 | | | P01344 |
| IL-10 | Interleukin-10 | | | | | 3586 | P22301 |
| IL-12p40 | Interleukin-12p40 | | | | | 3593 | P29460 |
| IL-18 | Interleukin-18 | | | | | 3606 | Q14116 |
| IL-27 | Interleukin-27 | | | | | 246778 | Q8NEV9 |
| IL-34 | Interleukin-34 | | | | | 146433 | Q6ZMJ4 |
| IL-4 | Interleukin-4 | | | | | 3565 | P05112 |
| LAG-3 | lymphocyte activation gene-3 | | | | | 3902 | P18627 |
| LEP | Leptin | | | | | 3952 | P41159 |
| LCN-2 | Lipocalin-2 | | | | | 3934 | P80188 |
| LTN | Lymphotactin | | | | | 6375 | P47992 |
| MCP-4 | monocyte chemoattractant protein-4 | | | | | 6357 | Q99616 |
| MIF | macrophage migration inhibitory factor | | | | | 4283 | Q07325 |
| MIP-1α | macrophage inflammatory protein-1α | | | | | 6348 | P10147 |
| MIP-3α | macrophage inflammatory protein-3α | | | | | 6364 | P78556 |
| MPIF-1 | myeloid progenitor inhibitory factor-1 | | | | | 6368 | P55773 |
| NCAM-1 | neural cell adhesion molecule-1 | | | | | 4684 | P13591 |
| PGRPs | peptidoglycan-recognition proteins | | | | | 8993 | 075594 |
| RANTES | regulated on activation,normal T cell expressed and secreted | | | | | 6352 | P13501 |
| SP-D | Pulmonary surfactant-associated protein-D | | | | | 66421 | P35247 |
| TLR2 | Toll like receptor 2 | | | | | 7097 | 060603 |
| TLR3 | Toll like receptor 3 | | | | | 7098 | 015455 |
| TSP-1 | thrombospondin-1 | | | | | 7057 | P07996 |
| VCAM-1 | vascular cell adhesion molecule-1 | | | | | 7412 | P19320 |
| ICAM-2 | intercellular adhesion molecule-2 | | | | | 3384 | P13598 |

*Data are presented as the Protein ID, Full name, Entrez ID and Uniport ID of 80 cytokines associated with immune and inflammatory responses of cytokine array analysis.*

**Supplementary Table 2. ROC analysis results of differentiation between anti-NMDAR encephalitis and viral encephalitis.**

| Cytokines and inflammatory mediators | Sensitivity | Specificity | Cutoff | AUC | 95%CI | *P* value |
| --- | --- | --- | --- | --- | --- | --- |
| cIAP-1  (ng/ml) | 1 | 0.692 | 66.37 | 0.834 | 0.672-0.997 | 0.004 |
| MCSF  (pg/ml) | 0.692 | 0.846 | 314.5 | 0.808 | 0.640-0.975 | 0.008 |
| CXCL13  (pg/ml) | 0.846 | 0.615 | 218.1 | 0.781 | 0.602-0.960 | 0.015 |
| NLRP3  (ng/ml) | 0.692 | 0.923 | 5.36 | 0.852 | 0.693-1 | 0.002 |

*Abbreviations: ROC, receiver operating characteristics; NMDAR, anti-N-methyl-D-aspartate receptor; CI, confidence interval; cIAP-1, Cellular inhibitor of apoptosis protein; MCSF, macrophage colony-stimulating factor; CXCL13, CXC chemokine ligand 13; NLRP3, nucleotide binding oligomerization domain—like receptor protein 3.*
